# Effect of high fat diet and excessive compressive mechanical force on pathologic changes of temporomandibular joint

**DOI:** 10.1038/s41598-020-74326-z

**Published:** 2020-10-15

**Authors:** Jing Du, Qian Jiang, Li Mei, Ren Yang, Juan Wen, Shuang Lin, Huang Li

**Affiliations:** 1 Department of Orthodontics, Nanjing Stomatological Hospital, Medical School of Nanjing University, Nanjing, 210008 Jiangsu People’s Republic of China; 2 Children’s Department of Stomatology, Stomatological Hospital of Chongqing Medical University, Chongqing, People’s Republic of China; 3 Discipline of Orthodontics, Department of Oral Sciences, Faculty of Dentistry, Sir John Walsh Research Institute, University of Otago, Dunedin, New Zealand

**Keywords:** Diseases, Risk factors

## Abstract

The aim of this study was to investigate the effect of high fat diet and excessive compressive mechanical force on temporomandibular joint. In vivo, a mouse model of temporomandibular joint compressive loading device was used. A high fat diet mouse model and a combined mouse model intraperitoneally treated with or without simvastatin were used in the study. The pathological changes of mandibular condylar cartilage were assessed by Safranin-O staining. The IL-1β, MMP-3, leptin expression changes in the cartilage were detected by immunohistochemistry. In vitro, the mandibular condylar chondrocytes were treated with or without L-1β and simvastatin. The mRNA expression level of matrix MMPs and leptin were assessed. Both excessive compressive mechanical force and high fat diet induced obesity caused TMJ osteoarthritis-like changes and increased expression of IL-1β, MMP-3, and leptin. These pathological changes were much more serious when the two interventions were exerted together, while simvastatin could obviously alleviate these changes. The mRNA expression of MMP-3, MMP-13, and leptin increased in the IL-1β treated chondrocytes treated with IL-1β, and decreased with simvastatin treatment. The development of temporomandibular joint pathological changes could be caused by the excessive compressive mechanical force and high fat diet induced obesity.

## Introduction

Temporomandibular joint osteoarthritis (TMJOA) is a multi-pathogenesis disease of increasing prevalence. There are many theories about its etiology, among which the exact mechanism of its pathological progress remains unclear. A variety of animal models have been constructed by different methods to investigate the pathogenesis of TMJOA, including surgery, mechanical force, chemical method and transgenic method. It has been found that the excessive mechanical force applied to a health articular cartilage and physiologic mechanical force applied to a damaged one could both result in the development of osteoarthritis^1–3^. In our previous study, a compressive mechanical force of 80 g, which was exerted upwards and backwards on the mandibular condyle of rat on both sides, caused a continuously retruded position of the mandible. It has been demonstrated that a long standing compressive mechanical force could cause a series of pathological changes inside the mandibular cartilage, including chondrocyte death, extracellular matrix degradation and reduced cartilage elasticity^4,5^. Even though these pathological changes have been reported to be regulated by endoplasmic reticulum stress and programmed cell death^2,6^, there might exist other mechanisms to be clarified.

Obesity is found to be associated with OA^7,8^. Being overweight changes the local mechanical stress, which may be an important reason for the onset and progress of OA in weight-bearing joints. However, this factor can’t explain the association between obesity and OA in non-weight-bearing joints such as hand, wrist and TMJ joints^9–11^. Furthermore, obesity is also associated with TMJOA according to some cross-sectional study^12,13^. On one hand, obesity might also induce TMJOA through increasing masticatory load, but this speculation needs further exploration. On the other hand, As a non-weight-bearing joint, obesity related TMJOA may be induced through some other unknown mechanisms besides mechanical stress change such as metabolic factors, so does excessive mechanical force related TMJOA. Since obesity is associated with mild, chronic inflammation^14^, over-expressed imflammatory factors and proinflammatory factors may play an important role in the mechanism of obesity related TMJOA. Leptin is one of the increased proinflammatory factors in people with obesity^15^. Leptin can also be synthesized by chondrocytes, synovial cells, bone cells and fat cells in the joint surrounding adipose tissue^16^. It has been found that patients with knee osteoarthritis have not only the increased level of serum leptin but also the abnormal leptin level in the synovial fluid^17^. Osteoarthritis has been increasingly considered as an inflammatory disease^18,19^, some studies have suggested that the elevated level of leptin and other fat factors can promote the synthesis of inflammatory factors (e.g., IL-1β, IL-6 and IL-8) and cartilage matrix degradation enzymes (e.g., MMP-9 and MMP-13) in knee joint that facilitate inflammatory response and cause active catabolic metabolism, resulting in cartilage matrix degradation^20,21^. Even though pathological changes in keen joint are similar to TMJOA, unlike hyaline cartilage in keen joint, fibrous cartilage in TMJ may have different pathogenesis when OA appears. Most of those previous studies of osteoarthritis were carried out on the knee joint, the association of obesity and excessive compressive mechanical force with TMJOA is still poorly understood. Does the lipid metabolic factor related mechanism also apply to obesity related TMJOA and excessive compressive mechanical force with TMJOA?

Statins is the present initial therapy for hyperlipidemia, among which simvastatin is one of the most commonly used. Statins have also been found to be effective in treating other diseases by inhibiting leptin expression^22–24^. Furthermore, it has been found that statins have a protective effect on the cartilage in the knee joint by promoting the proliferation and inhibiting the apoptosis of chondrocytes, as well as by inhibiting proinflammatory factors, suppressing MMPs and NO in chondrocytes^25–27^. However, for TMJOA caused by excessive compressive mechanical force and high fat diet, the therapeutic role of statins remains unclear.

The aim of the study was to investigate the effects of high fat diet and excessive compressive mechanical force on the development of pathological changes of the temporomandibular joint.

## Materials and methods

### Cell culture

Mandibular condylar chondrocytes were isolated from six 3-week-old male C57BL/6 mice considering the activity for passaging, and cultured to the third generation (P3) for the subsequent experiments as previously described in the literature^28^. The optimal concentration (1 μM) and time (6 h) of simvastatin treatment on the condylar chondrocytes were determined by cell counting kit-8 (CCK-8) (Supplementary Fig. 1). Then, the P3 cells were cultured in a low serum dulbecco's modified eagle medium (DMEM) containing 2% fetal bovine serum (FBS) and divided into four equivalent groups: the control, the ST group (treated with 1 μM simvastatin for 6 h), the IL-1β group (treated with 10 ng/ml IL-1β for 24 h), and the IL-1β + ST group (treated with both 10 ng/ml IL-1β for 24 h and 1 μM simvastatin for 6 h). The IL-1β treatment was executed at the 0 h and the same volume of PBS was added to other groups at the same time point. The simvastatin treatment was executed at the 18 h and the same volume of PBS was also added to other groups at the same time point. After a total treatment time of 24 h, all the four groups of cells were collected for RNA isolation and then quantitative determination.

### Animal models

Experimental protocols complied with the ARRIVE (Animal Research: Reporting in Vivo Experiments) guidelines for preclinical animal studies, and were approved by the Animal Care and Use Committee of Nanjing University. A total of 72 adult 8-week-old male C57BL/6 mice (weighed 20–25 g) were used in this study to build the following 3 different models (Supplementary Fig. 2). All animals were obtained from the Model Animal Research Center of Nanjing University, and housed in the specific-pathogen-free laboratory animal room at Nanjing University. All of the mice were acclimated to their surroundings for a day before the experiments.

### High fat diet mouse model

Twelve 8-week-old mice were randomly divided into normal diet (ND) (n = 6) and high fat diet (HFD) (n = 6) groups. The mice in HFD group were fed with diets containing 60 kcal% fat (D12492, Research Diets, America); the mice in ND group were fed with ordinary diets of identical hardness. They were weighed once a week and euthanized to acquire the temporomandibular joint (TMJ) specimens after 12 weeks of different diets; the weight gain trend was showed in Supplementary Fig. 3.

### Compressive mechanical force-applying mouse model

Twelve 8-week-old mice were randomly divided into the control (n = 6) and mechanical force (MF) (n = 6) groups. All of them were fed with normal diet. The mice in MF group were loaded with compressive mechanical force for 7 days^29^, simulating the device previously described on rats^2,30^. Briefly, two hooks were made of 0.025-in. stainless steel wire on each lateral side of the cleaned lower incisors of the mice; then, the resin was shaped into a sphere to stop the hooks from coming out as an undercut. The copper wire made anchorage jigs were placed around a mouse's neck and arms. On each side, a compressive mechanical force was loaded upward and backward by a rubber band tied between the anchorage jig and the hook. The force value was a quarter of that exerted on a rat according to the volume ratio of temporomandibular joints in the mouse and rat. None of the mice displayed signs of disability. The detailed process was shown in Supplementary Fig. 4.

### Combined mouse model

Forty-eight 8-week-old mice were randomly divided into eight groups: normal diet (ND) (n = 6), high fat diet (HFD) (n = 6), normal diet with mechanical force (ND + MF) (n = 6), mechanical force with high fat diet (HFD + MF) (n = 6), normal diet with statin (ND + ST) (n = 6), high fat diet with simvastatin (HFD + ST) (n = 6), normal diet with mechanical force and simvastatin (ND + MF + ST) (n = 6), and mechanical force with high fat diet and simvastatin (HFD + MF + ST) (n = 6).

All mice in groups of ND and HFD were fed with different diets as the methods described above for 2 weeks before the beginning of the experiment until they were euthanized. The mice in groups of MF were applied with mechanical force for 7 days referring to the procedures above. The mice in groups of ST were treated with simvastatin (ProSpec, Israel) transperitoneally at a dose of 40 mg/kg/d^31^ for 5 consecutive days from the third day of force-applying, while the others were treated with a same volume of PBS for the same time as control.

### Quantitative real-time polymerase chain reaction (qRT-PCR)

The primers of MMP-3, MMP-13, leptin and glyceraldehyde-3-phosphate dehydrogenase (GAPDH) for qRT-PCR are listed in Supplementary Table 1. The PCR were performed in a 10 μl mixture using a 2 × SYBR real-time PCR premixture kit. The 2^−ΔΔCT^ method was used to calculate the amount of target cDNA relative to GAPDH.

### Tissue preparation and histological analysis

The TMJ tissues of the mice were prepared as we have previously described^2^. Three slides of central sagittal sections from each sample were chosen for safranin-O staining to assess the histopathological changes of TMJ.

All images were captured by an Olympus XI 70 microscope equipped with an Olympus Magna Fire digital camera. The thickness of the mandibular condylar cartilage was measured according to the measuring scale by a single observer who was blinded to the experimental protocol. The condylar cartilage was divided into 3 layers from outside to inside as shown in Supplementary Fig. 5: the fibrous layer, proliferative layer, and hypertrophic/calcified cartilage layer^32^. We measured the thickness of the three layers respectively and the total thickness. We measured in 3 squares located at the central third of the cartilage. The mean value of the 3 squares in each slide was used for further statistical analysis. We also scored the sections according to a semi-quantitative scoring system as described in Supplementary Table 2^33^, which is characterized by the mount of matrix loss and extent of vertical cleft in the cartilage. However, in the mechanical force-applying mouse model and combined mouse model, we couldn’t refer to this scoring system because the experiment period the mice could bear was not long enough to cause obvious vertical cleft on the surface of cartilage.

### Immunohistochemistry

Immunohistochemistry was performed according to standard protocols to detect the expression of IL-1β, MMP-3, MMP-13 and leptin. In brief, 5-μm–thick sections (3 sections per specimen) were prepared according to standard protocol for Safranin-O staining. Rabbit anti-IL-1β (1:100; Abcam), Rabbit anti-MMP-3 (1:200; Abcam), Rabbit anti-MMP-13 (1:100; Abcam), Rabbit anti-leptin (1:150; Abcam) served as the primary antibodies. Goat anti-rabbit horseradish peroxidase-conjugated IgG was used as the secondary antibody.

The immunohistochemical measurements were made by 2 observers and the differences between observers were reconciled by microscope conferencing. The sections were assessed as the intensity of the stain in the condylar cartilage (0, negative, no staining; 1, faint yellow, mild staining; 2, clay bank, moderate staining; 3, brown, intense staining).

### Statistical analysis

The data are expressed as means ± standard deviation (SD), and the statistical analysis was conducted using one-way ANOVA for histological assessment and qRT-PCR and Mann–Whitney *U* nonparametric statistical test for immunohistochemistry. All data were analyzed with Statistic Package for Social Science (SPSS) 18.0 software. The differences between treatment groups were considered significant when *P* < 0.05.

## Results

### The mRNA expression level of leptin increased in the IL-1β treated chondrocytes

**Figure 1:**
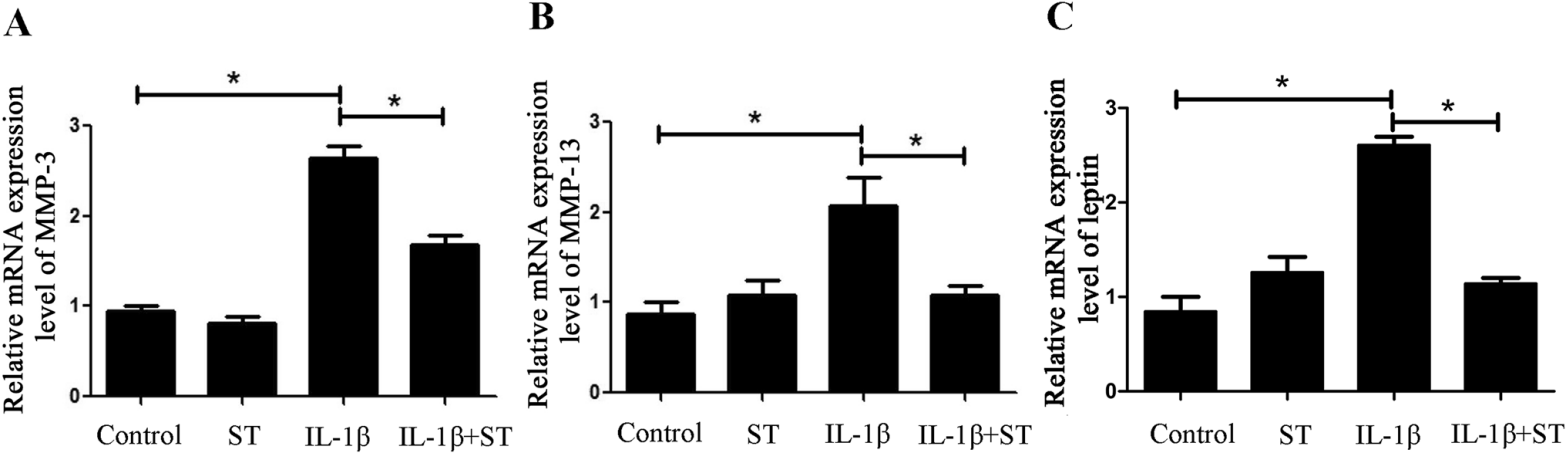
IL-1β up-regulated the mRNA expression level of MMP-3, MMP-13 and leptin, while simvastatin alleviated these phenomena. (**A**) MMP-3 mRNA expression levels of condylar chondrocytes treated with or without IL-1β and simvastatin. (**B**) MMP-13 mRNA expression levels of condylar chondrocytes treated with or without IL-1β and simvastatin. (**C**) Leptin mRNA expression level of condylar chondrocytes treated with or without IL-1β and simvastatin. *P < 0.05.

After 24 h of IL-1β treatment on the condylar chondrocytes, the mRNA expression level of cartilage matrix degradation enzyme, MMP-3 and MMP-13, increased by 170% (Fig. 1A) and 130% (Fig. 1B) respectively, and the mRNA expression of leptin was also up-regulated by 150% compared with Control (Fig. 1C). However, after 6 h of simvastatin treatment, the cells in IL-1β + ST group expressed leptin mRNA almost at a normal level compared with Control (Fig. 1C). Correspondingly, the mRNA expression of MMP-3 and MMP-13 in the IL-1β + ST group decreased by 37% (Fig. 1A) and 48% (Fig. 1B) respectively compared with the IL-1β group. These results suggested that condylar chondrocytes under inflammation showed higher matrix degradation activity and expressed more leptin. However, when the leptin expression was down-regulated by simvastatin, the expression of cartilage matrix enzymes dropped.

### Long time of high fat diet caused TMJOA-like lesions and up-regulated leptin expression in mandibular condylar cartilage

**Figure 2:**
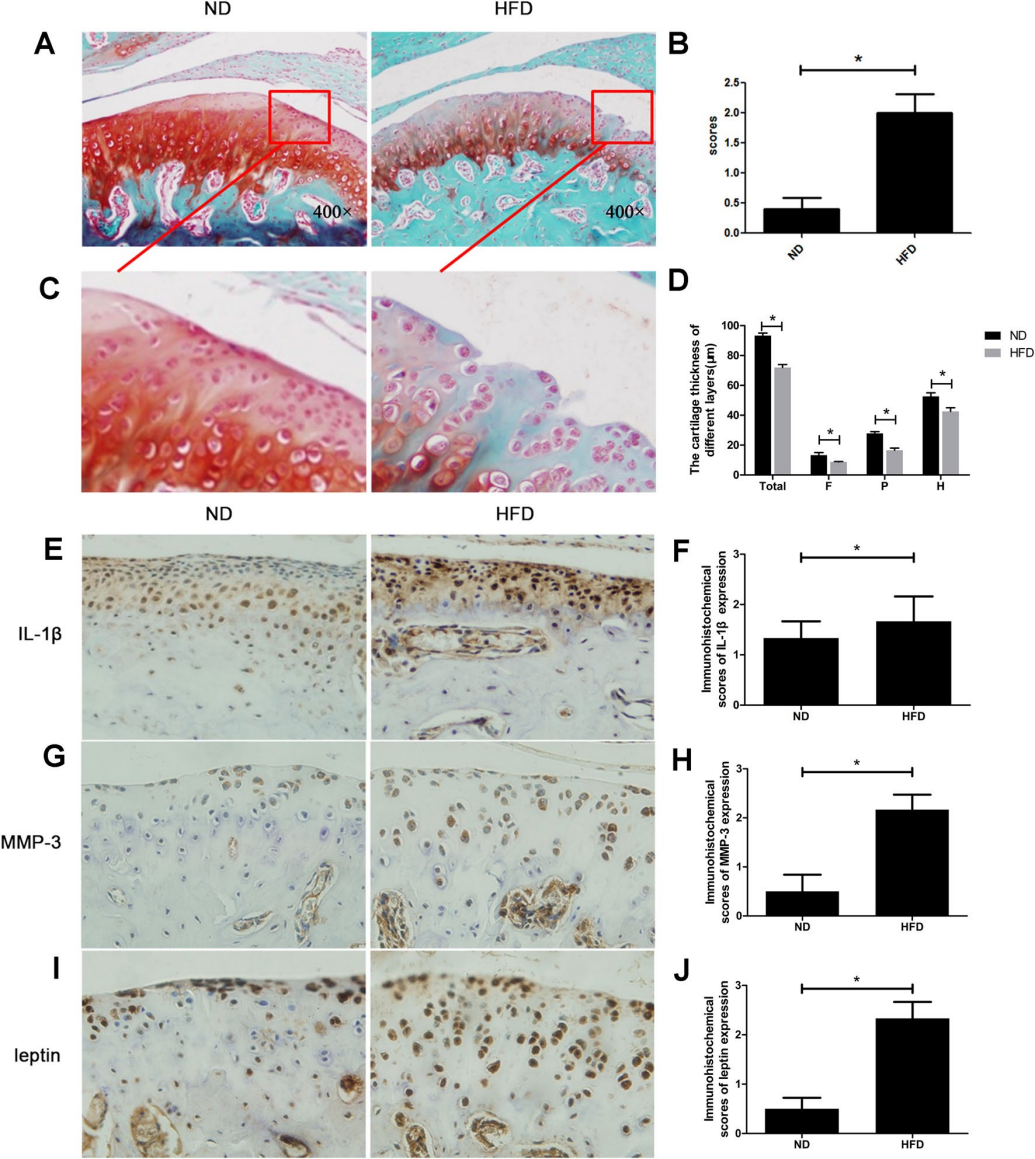
The HFD mice got TMJOA-like pathological changes and increased leptin expression compared to ND mice. (**A**) Histological morphology of the condylar cartilage of ND mice and HFD mice (400 ×). (**B**) Relative scores of HFD mice and ND mice. (**C**) Magnified local regions of condylar cartilage in ND mice and HFD mice. (**D**) Cartilage thickness of different layers in ND mice and HFD mice (*T* total layer, *F* fibrous layer, *P* proliferative layer, *H* hypertrophic layer). (**E**). Expression of IL-1β in the condylar cartilage of HFD mice and ND mice. (**F**) Immunohistochemical scores of IL-1β in ND and HFD groups. (**G**) Expression of MMP-3 in the condylar cartilage of HFD mice and ND mice. (**H**) Immunohistochemical scores of MMP-3 in ND and HFD groups. (**I**) Leptin expression in the condylar cartilage of HFD mice and ND mice. (**J**) Immunohistochemical scores of leptin in ND and HFD groups. *P < 0.05.

Mice in the ND group generally maintained normal morphology of condylar cartilage and normal quantities of cartilage matrix which was stained red, while overweight mice in the HFD group showed morphological changes of condylar cartilage and less cartilage matrix (Fig. 2A). HFD mice had 23% thinner condylar cartilage than ND mice (Fig. 2D). There appeared vertical clefts of different depth in the superficial layer (marked in red rectangle in 2A and magnified in Fig. 2C) of HFD mice’s condylar cartilage. According to the semi-quantitative scoring system, HFD group scored 450% higher than ND group statistically (Fig. 2B), which means severer pathological changes of cartilage in HFD mice. The immunohistochemistry results showed that the expression of inflammatory factor IL-1β and cartilage matrix degradation enzyme MMP-3 were up-regulated in the condylar cartilage of HFD mice by 25% and 330% compared to ND mice (Fig. 2E–H), indicating that HFD mice got TMJOA-like pathological changes. We also detected that the expression of leptin was up-regulated by 367% (Fig. 2I,J), suggesting that leptin might play an important role in the pathogenetic process of high fat diet caused TMJOA-like changes.

### Compressive mechanical force caused TMJOA-like pathological changes and leptin expression increased in condylar cartilage

**Figure 3:**
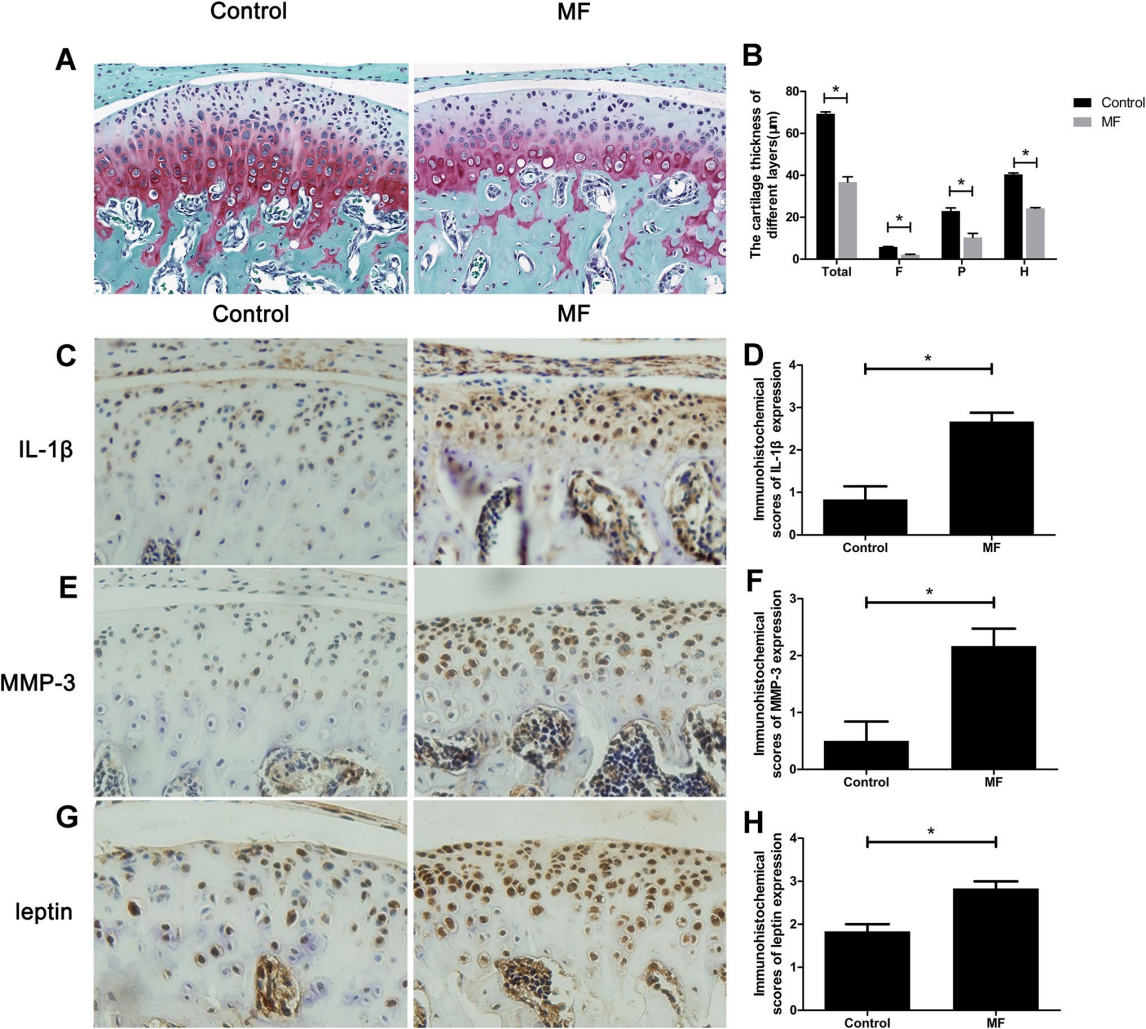
The MF mice got TMJOA-like pathological changes and increased leptin expression compared to Control. (**A**) The histological morphology of the condylar cartilage of MF mice and Control (400 ×). (**B**) The cartilage thickness of different layers in MF mice and Control (T: total layer; F: fibrous layer; P: proliferative layer; H: hypertrophic layer). (**C**) The expression of inflammatory factor, IL-1β in the condylar cartilage of MF mice and Control. (**D**) Immunohistochemical scores of IL-1β after force applying for 7 days. (**E**) The expression of cartilage matrix degradation enzyme, MMP-3 in the condylar cartilage of MF mice and Control. (**F**) Immunohistochemical scores of MMP-3 after force applying for 7 days. (**G**) Leptin expression in the condylar cartilage of MF mice and Control. (**H**) Immunohistochemical scores of leptin after force applying for 7 days. *P < 0.05.

The results of safranin-O staining showed that the condylar cartilage of the mice in MF group got damaged. After applied with compressive mechanical force for 7 days, the condylar cartilage became thinner by 47% (Fig. 3B), and the amount of cartilage matrix was less (Fig. 3A) than those of Control. The immunohistochemistry results also showed increased IL-1β (Fig. 3C,D), MMP-3 (Fig. 3E,F), and leptin (Fig. 3G,H) expression level in the condylar cartilage of MF mice by 220%, 30%, and 55% respectively, indicating that the mechanical force induced TMJOA-like changes were also related to leptin expression increase in the condylar cartilage, which was consistent with the results in HFD mouse model. However, the reduction of condylar cartilage thickness was more notable in MF mice compared to HFD mice, while the elevation of leptin was more remarkable in HFD mice when compared with MF mice.

### Compressive mechanical force induced pathological changes were exacerbated by high fat diet and alleviated by simvastatin

**Figure 4:**
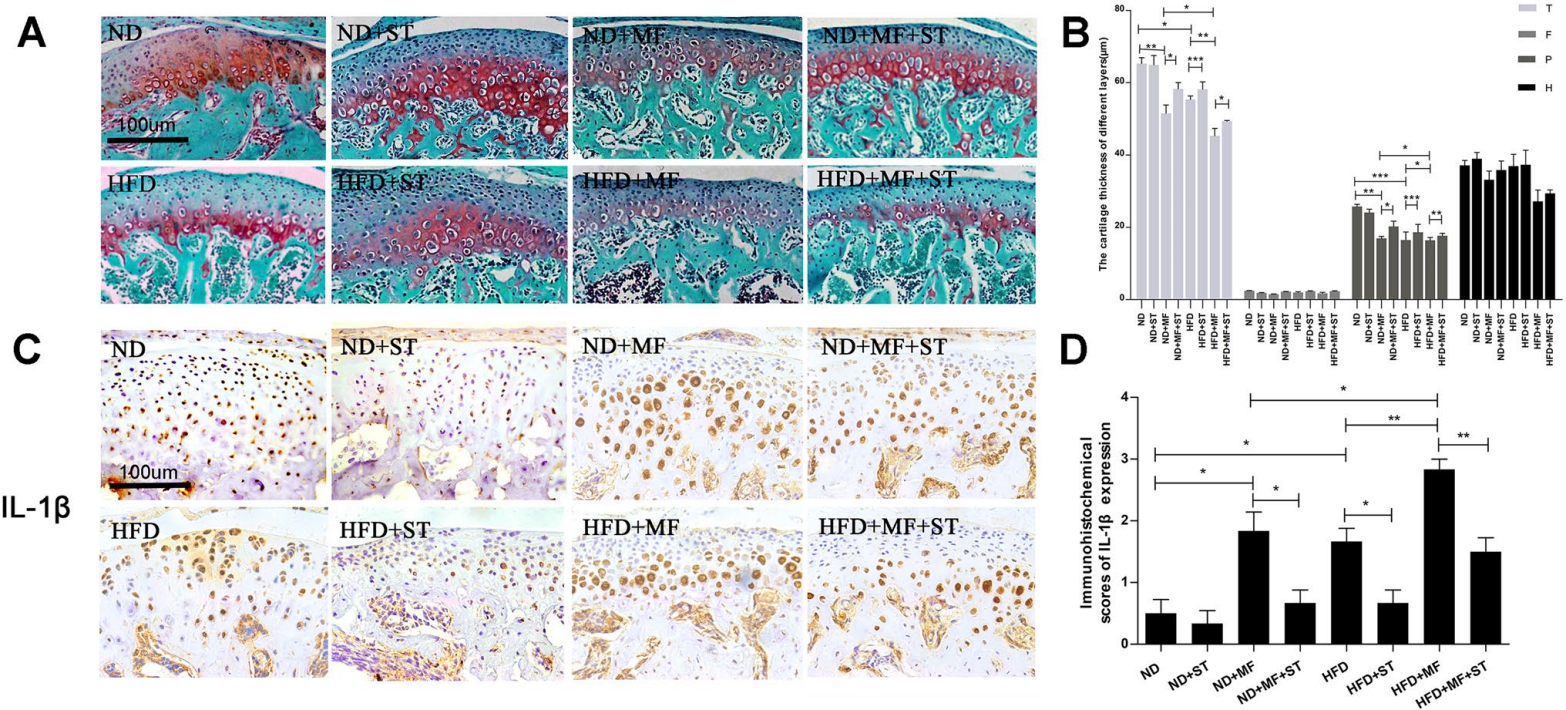
Mechanical force induced TMJOA-like damages were exacerbated by high fat diet and alleviated by simvastatin. (**A**) The histological morphology of condylar cartilage of the mice in the eight different groups of ND, ND + ST, ND + MF, ND + MF + ST, HFD, HFD + ST, HFD + MF, HFD + MF + ST. (400 ×). (**B**) The cartilage thickness of different layers in the mice of the eight different groups (*T* total thickness, *F* fibrous layer, *P* proliferative layer, *H* hypertrophic layer). (**C**) The expression of inflammatory factor, IL-1β in the condylar cartilage of the mice in the eight different groups. (**D**) Immunohistochemical scores of IL-1β in the eight different groups. *P < 0.05; **P < 0.01.

**Figure 5:**
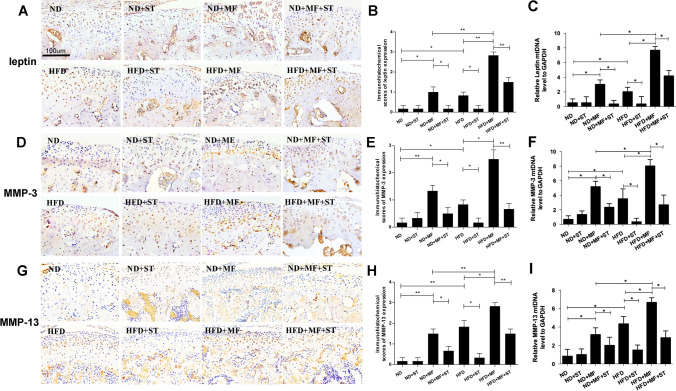
(**A**) Leptin expression in the condylar cartilage of the mice in the eight different groups. (**B**) Immunohistochemical scores of leptin in the eight different groups. (**C**) Leptin mRNA expression level in the condylar cartilage of the eight goups. (**D**) The expression of cartilage matrix degradation enzyme, MMP-3 in the condylar cartilage of the mice in the eight different groups. (**E**) Immunohistochemical scores of MMP-3 in the eight different groups. (**F**) MMP-3 mRNA expression level in the condylar cartilage of the eight groups. (**G**) The expression of cartilage matrix degradation enzyme, MMP-13 in the condylar cartilage of the eight different groups. (**H**) Immunohistochemical scores of MMP-13 in the eight different groups. (**I**) MMP-13 mRNA expression level in the condylar cartilage of the eight groups. *P < 0.05; **P < 0.01.

In the experiment using the combined mouse model, the results of safranin-O clearly showed the morphological changes when the mice were fed with high fat diet, applied with compressive mechanical force, or injected with simvastatin afterwards. The mice with normal diet and applied with mechanical force for 7 days (ND + MF) got thinner condylar cartilage, irregularly arranged chondrocytes and reduced condylar cartilage matrix, which was consistent with our results above. However, the identical mechanical force applied on the mice fed with high fat diet (HFD + MF) achieved much more serious histological damages with 12% thinner cartilage and less cartilage matrix compared to ND + MF mice (Fig. 4A,B). The immunohistochemistry results also showed aggravated over-expression of IL-1β, MMP-3, MMP-13 and leptin in the condylar cartilage of the mice in HFD + MF group when compared with those in ND + MF group by 55%, 88%, 88% and 183% respectively (Figs. 4C,D, 5A,B,D,E,G,H). These results suggested that compressive mechanical force induced pathological changes could be exacerbated by high fat diet. The qPCR results showed similar changes. The mRNA expression of MMP-3, MMP-13 and leptin in HFD + MF group were increased significantly when compared with that of ND + MF group (Fig. 5C,F,I). However, after 5 consecutive days of intraperitoneal injection with simvastatin, the histological damages described above were alleviated (Fig. 4A,B). We scored the safranin staining part which stands for the proteoglycan content following the method of immunohistochemistry evaluation. the results were in line with expectation (data not shown). The cartilage thickness augment were 5% in HFD + ST group compared to HFD group, 13% in ND + MF + ST group compared to ND + MF group, and 9% in HFD + MF + ST group compared to HFD + MF group. Even though the cartilage thickness improvement seemed not that remarkable, which might due to our relatively short experiment period, we found that simvastatin made a real difference on the expression of leptin, inflammatory factor (IL-1β) and matrix proteolytic enzyme (MMP-3, MMP-13). After the simvastatin treatment, the abnormally increased leptin, IL-1β, MMP-3 and MMP-13 expression were notably down-regulated (Figs. 4C,D, 5A,B,D,E,G,H). In the HFD + ST group, IL-1β, MMP-3, MMP-13 and leptin expression decreased by 60%, 80%, 82% and 80% respectively, compared to HFD group. In the ND + MF + ST group, IL-1β, MMP-3, MMP-13 and leptin expression decreased by 64%, 63%, 56% and 83% respectively, compared to ND + MF group. In the HFD + MF + ST group, IL-1β, MMP-3, MMP-13 and leptin expression decreased by 47%, 73%, 53% and 47% respectively, compared to HFD + MF group. The qPCR results also showed that the changes on mRNA level were in line with that on protein level detected by immunohistochemistry. (Fig. 5C,F,I) The alleviating effects of simvastatin on the pathological changes we found in vivo were consistent with the results in vitro described above.

## Discussion

The obesity has become increasingly prevalent. There have been numerous studies showing that the occurrence of obesity has positive correlation with the incidence of osteoarthritis in knee^34^, which was originally thought to be caused by the long-term excessive load on knee joint because of obese people’s weight gain^7,8^. In this study, we found that there exists similar correlation between obesity and osteoarthritis in a non-heavy-load joint, TMJ. The result showed that the obese mice with high fat diets got TMJOA-like changes in the condylar cartilage, including morphological changes, up-regulated expression of inflammatory factor, cartilage matrix degradation enzyme and excessive leptin expression. In the other hand, the similar TMJOA-like pathological changes induced by excessive compressive mechanical force on mice was also demonstrated in this study, and the high fat diet induced obesity exacerbated the TMJOA-like changes induced by excessive compressive mechanical force. All these results suggested that obesity is one of the risk factors of TMJOA.

Statins is clinically used as the initial therapy for blood lipid disorders presently. As researches further develop, statins have been found to have therapeutic effect on a lot of other diseases besides reducing blood lipid. There has been study showing that statins can reduce the risk of cognitive impairment, dementia, fractures, venous thrombosis, and pneumonia^35^. Moreover, some studies discuss about the treatment of osteoarthritis by statins, which is found to be able to promote the proliferation and inhibit the apoptosis of chondrocytes in the knee joint^26^, as well as delaying the chondrocyte aging and cartilage matrix degradation in the knee joint and the hip joint^27^. In TMJ, George et al.^36^ reported that statins had effects of anti-inflammation to the rats suffered TMJOA, and Holwegner et al.^37^ found that simvastatin can maintain the normal growth of the subchondral bone in the condyle of rats suffered TMJOA. In this study, we confirmed that statins had effects of anti-inflammation in TMJOA-like changes and it had a protective effect on the damaged TMJ cartilage. Thus, we think that statins may have a potential therapeutic effect on TMJOA. For TMJOA patient with obesity, statins may be used to treat hyperlipidemia as lipid-lowering drug and also have protective effect on the TMJ. But for TMJOA patient with no obesity, the side effects of statins should be noted. Further studies need to do in the future before it can be put into clinic.

In addition, excessive leptin expression was detected in the condylar chondrocytes under inflammation in vitro in the study. Leptin is one of the fat factors which have proinflammatory activity. It can induce the synthesis of other proinflammatory factors and cytokines, and further promote the synthesis of proteoglycan enzymes and matrix metalloproteinases that participate in the pathological process of knee OA^20,38^. In this study, excessive leptin expression was also found in the mice with obesity and excessive mechanical force induced TMJOA-like changes, especially in HFD mice, suggesting that this proinflammatory effect of leptin may also promote the development of TMJOA. Meanwhile, Maeda et al.^39^ found that the leptin expression of preadipocytes was inhibited when using statins to stimulate the cells. Consistently, in this study, when simvastatin was used, the leptin expression was down-regulated both in vitro and in vivo, and the TMJOA-like changes were alleviated. All these results indicated that fat factor, specifically leptin, might be the mechanism of obesity related TMJOA and excessive mechanical force related TMJOA, and the protective effect on the TMJOA-like changes of simvastatin was exerted by inhibiting leptin expression. Our study opened up a new perspective of metabolic disorder for the research on TMJOA pathogenesis and provided a new potential target for the treatment of TMJOA. However, since simvastatin is a lipid-lowring drug, Simvastatin may not only have directly effects of anti-inflammation in mechanical force groups and high fat diet groups by inhibiting leptin expression, but also have effects of lipid-lowering in the high fat diet group, which may be associated with reduced weight and contribute to the protective effect.

There still exist some limitation in our study. For instance, we used only male mice for examination considering that female hormones might have interference with the results, but female animals should also be involved in order to get more comprehensive conclusion in animal experiment stage. Furthermore, a large number of studies need to be conducted to confirm the protective effect and to explore the mechanism of simvastatin treatment on TMJOA-like changes. After that, further clinical trials are needed to demonstrate if statins can be a potential drug for TMJOA clinically.

## Conclusion

Both excessive compressive mechanical force and high fat diet induced obesity could cause temporomandibular joint pathological changes. Metabolic disorder might played an important role in the pathogenetic process of TMJOA-like changes. A healthy diet minimizing obesity might be beneficial for the prevention of TMJOA and statins might be a potential pharmacotherapy for TMJOA.

## Supplementary information

Supplementary Informations.

## References

[1] Tanaka E, Detamore MS, Mercuri LG (2008). Degenerative disorders of the temporomandibular joint: etiology, diagnosis, and treatment. J. Dent. Res..

[2] Li H (2013). Endoplasmic reticulum stress regulates rat mandibular cartilage thinning under compressive mechanical stress. J. Biol. Chem..

[3] Li H (2010). Proteomic analysis of early-response to mechanical stress in neonatal rat mandibular condylar chondrocytes. J. Cell. Physiol..

[4] Zhang C, Xu Y, Cheng Y, Wu T, Li H (2015). Effect of asymmetric force on the condylar cartilage, subchondral bone and collagens in the temporomandibular joints. Arch. Oral Biol..

[5] Jiang YY (2017). BIO Allieviated compressive mechanical force-mediated mandibular cartilage pathological changes through Wnt/β-catenin signaling activation. J. Orthopaedic Rese..

[6] Huang Z (2017). Mechanical and hypoxia stress can cause chondrocytes apoptosis through over-activation of endoplasmic reticulum stress. Arch. Oral Biol..

[7] Widmyer MR (2013). High body mass index is associated with increased diurnal strains in the articular cartilage of the knee. Arthritis Rheum..

[8] Yusuf E (2009). Association between weight or body mass index and hand osteoarthritis: a systematic review. Osteoarthr. Cartil..

[9] Oliveria SA, Felson DT, Cirillo PA, Reed JI, Walker AM (1999). Body weight, body mass index, and incident symptomatic osteoarthritis of the hand, hip, and knee. Epidemiology.

[10] Carman WJ, Sowers MF, Hawthorne VM, Weissfeld LA (1994). Obesity as a risk factor for osteoarthritis of the hand and wrist: a prospective study. Am. J. Epidemiol..

[11] Cicuttini FM, Baker JR, Spector TD (1996). The association of obesity with osteoarthritis of the hand and knee in women: a twin study. J. Rheumatol..

[12] Jordani PC (2017). Obesity as a risk factor for temporomandibular disorders. J. Oral. Rehabil..

[13] Karaman A, Sadry S (2019). Evaluation of temporomandibular disorders and oral health-related quality of life with obese patients. Cranio.

[14] Wellen KE, Hotamisligil GS (2005). Inflammation, stress, and diabetes. J. Clin. Invest..

[15] Powell A, Teichtahl AJ, Wluka AE, Cicuttini FM (2005). Obesity: a preventable risk factor for large joint osteoarthritis which may act through biomechanical factors. Br. J. Sports Med..

[16] Scotece M (2013). Adipokines as drug targets in joint and bone disease. Drug Discov. Today.

[17] Conde J (2013). Adipokines: novel players in rheumatic diseases. Discov. Med..

[18] Berenbaum F (2013). Osteoarthritis as an inflammatory disease (osteoarthritis is not osteoarthrosis!). Osteoarthr. Cartil..

[19] Shen M (2013). 1,25(OH)2D deficiency induces temporomandibular joint osteoarthritis via secretion of senescence-associated inflammatory cytokines. Bone.

[20] Yang WH (2013). Leptin induces IL-6 expression through OBRl receptor signaling pathway in human synovial fibroblasts. PLoS ONE.

[21] Simopoulou T (2007). Differential expression of leptin and leptin's receptor isoform (Ob-Rb) mRNA between advanced and minimally affected osteoarthritic cartilage; effect on cartilage metabolism. Osteoarthr. Cartil..

[22] Sun YM, Li J, Luan Y, Wang LF (2010). Effect of statin therapy on leptin levels in patients with coronary heart disease. Peptides.

[23] Takahashi Y, Satoh M, Tabuchi T, Nakamura M (2012). Prospective, randomized, single-blind comparison of effects of 6 months' treatment with atorvastatin versus pravastatin on leptin and angiogenic factors in patients with coronary artery disease. Heart Vessels.

[24] Gholamin S (2014). Lovastatin for reduction of leptin in nondialysis patients with type 2 diabetic nephropathy. Iran J. Kidney Dis..

[25] Zhou B, Chen D, Xu H, Zhang X (2017). Proliferation of rabbit chondrocyte and inhibition of IL-1β-induced apoptosis through MEK/ERK signaling by statins. Vitro Cell. Dev. Biol. Anim..

[26] Bayyurt S, Bilgen MS, Bilgen ÖF, Yalçınkaya U (2015). The chondroprotective effects of intraarticular application of statin in osteoarthritis: an experimental study. Indian J. Orthop..

[27] Lazzerini PE (2004). Simvastatin reduces MMP-3 level in interleukin 1beta stimulated human chondrocyte culture. Ann. Rheum. Dis..

[28] Bordin TB, Conci RA, Pezzini MM, Pezzini RP, Mendonca MJ (2013). Prevalence of signs and symptoms of temporomandibular disorders (TMD) in patients wearing bimaxillary complete dentures, removable partial dentures and in students with natural dentition. Acta Odontol. Latinoam. AOL.

[29] Zhu M, Zhou S, Huang Z, Wen J, Li H (2016). Ca2+-dependent endoplasmic reticulum stress regulates mechanical stress-mediated cartilage thinning. J. Dent. Res..

[30] Teramoto M, Kaneko S, Shibata S, Yanagishita M, Soma K (2003). Effect of compressive forces on extracellular matrix in rat mandibular condylar cartilage. J. Bone Miner. Metab..

[31] McKay A, Leung BP, McInnes IB, Thomson NC, Liew FY (2004). A novel anti-inflammatory role of simvastatin in a murine model of allergic asthma. J. Immunol..

[32] Fujita T (2006). Influence of sex hormone disturbances on the internal structure of the mandible in newborn mice. Eur. J. Orthod..

[33] Glasson SS, Chambers MG, Van Den Berg WB, Little CB (2010). The OARSI histopathology initiative—recommendations for histological assessments of osteoarthritis in the mouse. Osteoarthr. Cartil..

[34] Hart DJ, Spector TD (1993). The relationship of obesity, fat distribution and osteoarthritis in women in the general population: the Chingford Study. J. Rheumatol..

[35] Macedo AF (2014). Unintended effects of statins from observational studies in the general population: systematic review and meta-analysis. BMC Med..

[36] George MD (2013). Effect of simvastatin injections on temporomandibular joint inflammation in growing rats. J. Oral Maxillofac. Surg..

[37] Holwegner C, Reinhardt AL, Schmid MJ, Marx DB, Reinhardt RA (2015). Impact of local steroid or statin treatment ofexperimental temporomandibular joint arthritis on bone growth in young rats. Am. J. Orthod. Dentofac. Orthop..

[38] Toussirot E, Streit G, Wendling D (2007). The contribution of adipose tissue and adipokines to inflammation in joint diseases. Curr. Med. Chem..

[39] Maeda T, Horiuchi N (2009). Simvastatin suppresses leptin expression in 3T3-L1 adipocytes via activation of the cyclic AMP-PKA pathway induced by inhibition of protein prenylation. J. Biochem..

