# Supplementary material for: Effect of high fat diet and excessive compressive mechanical force on pathologic changes of temporomandibular joint
Source: Sci Rep. 2020 Oct 15;10:17457. doi: 10.1038/s41598-020-74326-z (PMC7566592; doi:10.1038/s41598-020-74326-z)
Supplement: Supplementary file 1 — Supplementary Informations. [file 41598_2020_74326_MOESM1_ESM.docx]

**Effect of High Fat Diet and Excessive Compressive Mechanical Force on Pathologic Changes of Temporomandibular Joint**

Jing Du^1, 2, *^, Qian Jiang^1, *^, Li Mei^3^, Ren Yang^1^, Juan Wen^1^, Shuang Lin^1^, Huang Li^1^

1. Department of Orthodontics, Nanjing Stomatological Hospital, Medical School of Nanjing University, Nanjing, Jiangsu, People's Republic of China.

2. Children's department of stomatology, Stomatological Hospital of Chongqing Medical University, ChongQing, People's Republic of China.

3. Discipline of Orthodontics, Department of Oral Sciences, Sir John Walsh Research Institute, Faculty of Dentistry, University of Otago, Dunedin, New Zealand.

^*^: These authors contributed equally to this work

Correspondence: Huang Li

Tel: 0086 -25-83620173; Fax: 0086-25-83620173;

E-mail: [Lihuang76@nju.edu.cn](mailto:Lihuang76@nju.edu.cn),

Address: Department of Orthodontics, Nanjing Stomatological Hospital, Medical School of Nanjing University, Nanjing, Jiangsu, People's Republic of China, 210008.

**Supplementary Materials**


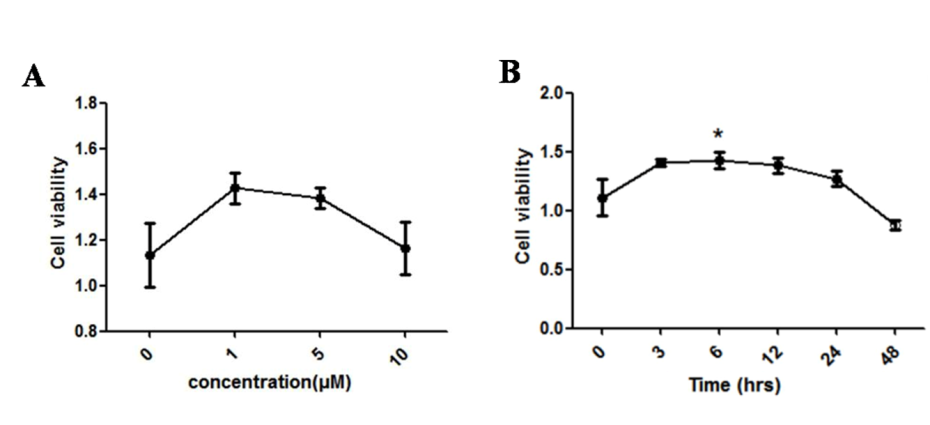


**Supplementary Figure 1**. The optimal concentration (1μM) and time (6h) of simvastatin treatment on the condylar chondrocytes. (A) The curve of cell viability with different concentrations of simvastatin treatment. (B) The curve of cell viability for different time periods of simvastatin treatment.

**
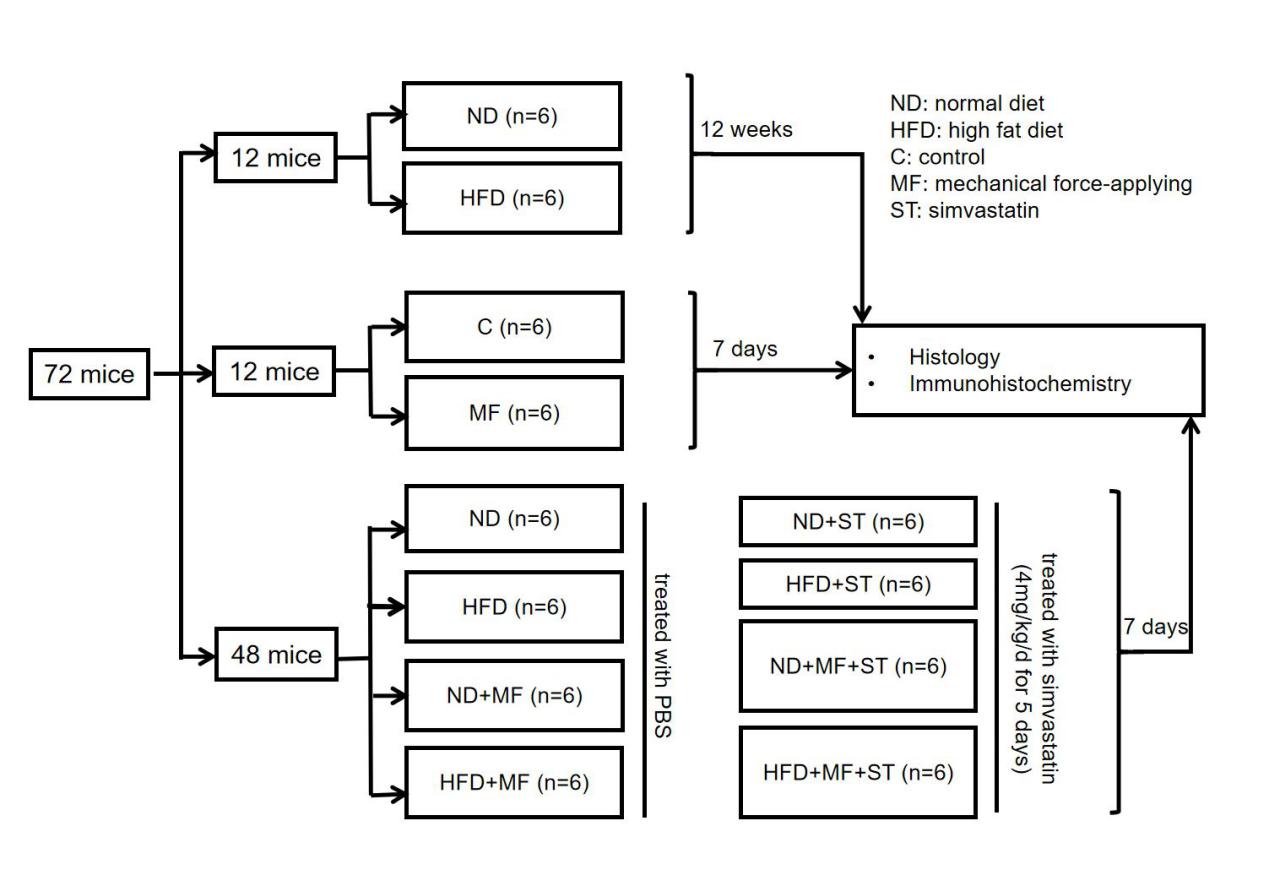
**

**Supplementary Figure 2.** Flowing chart showing the experimental design of the animal studies


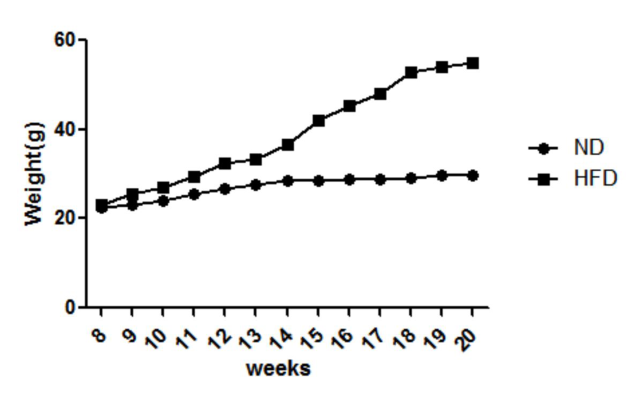


**Supplementary Figure 3.** The weight gain trend of HFD (high fat diet) mice and ND (normal diet) mice during 12 weeks.


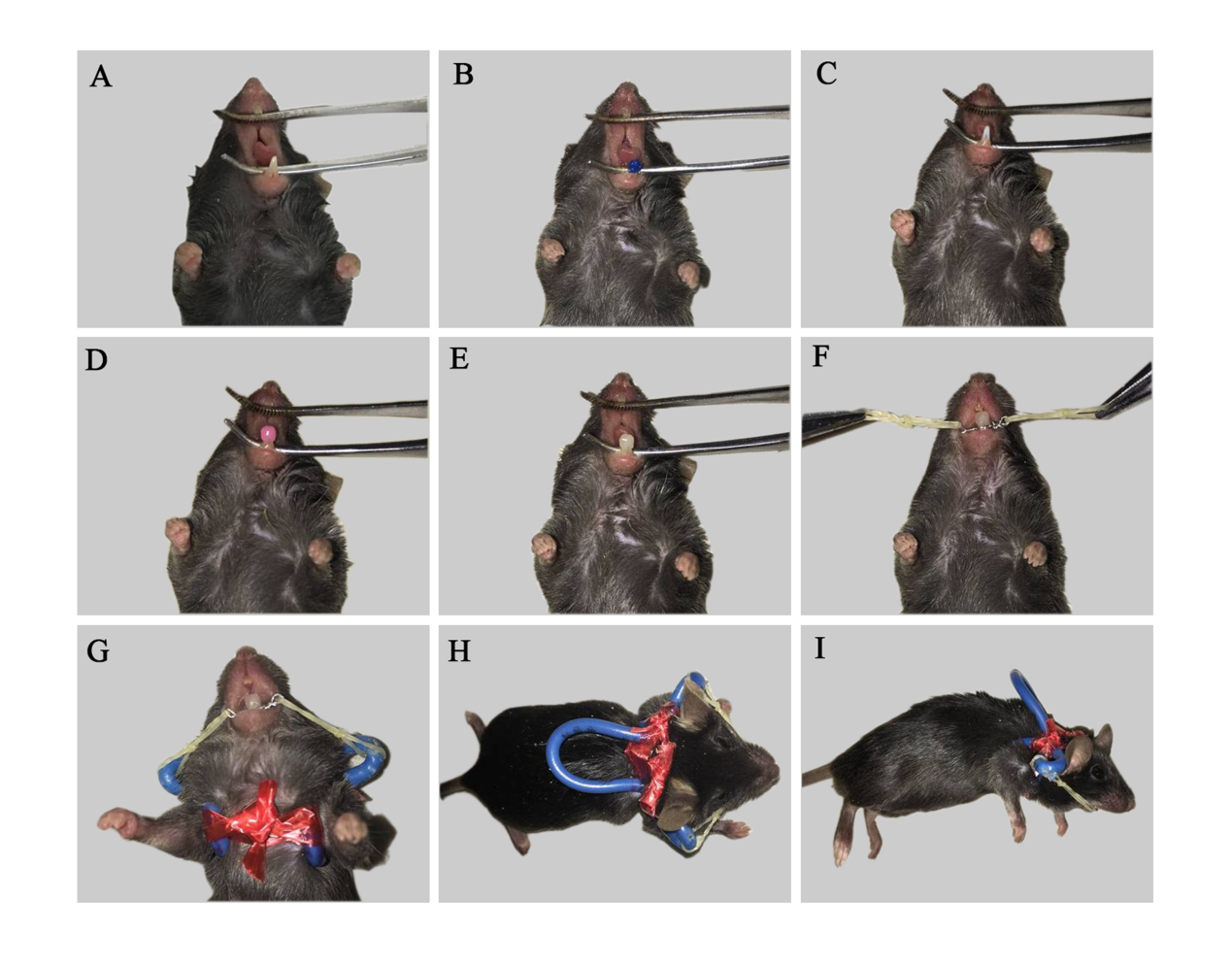


**Supplementary Figure 4.** The detailed process of building mechanical force-applying mouse model. (A) mouth opening; (B) acid etching; (C) drying; (D) spherical resin; (E) light curing; (F) rubber band installing; (G to I) finished.


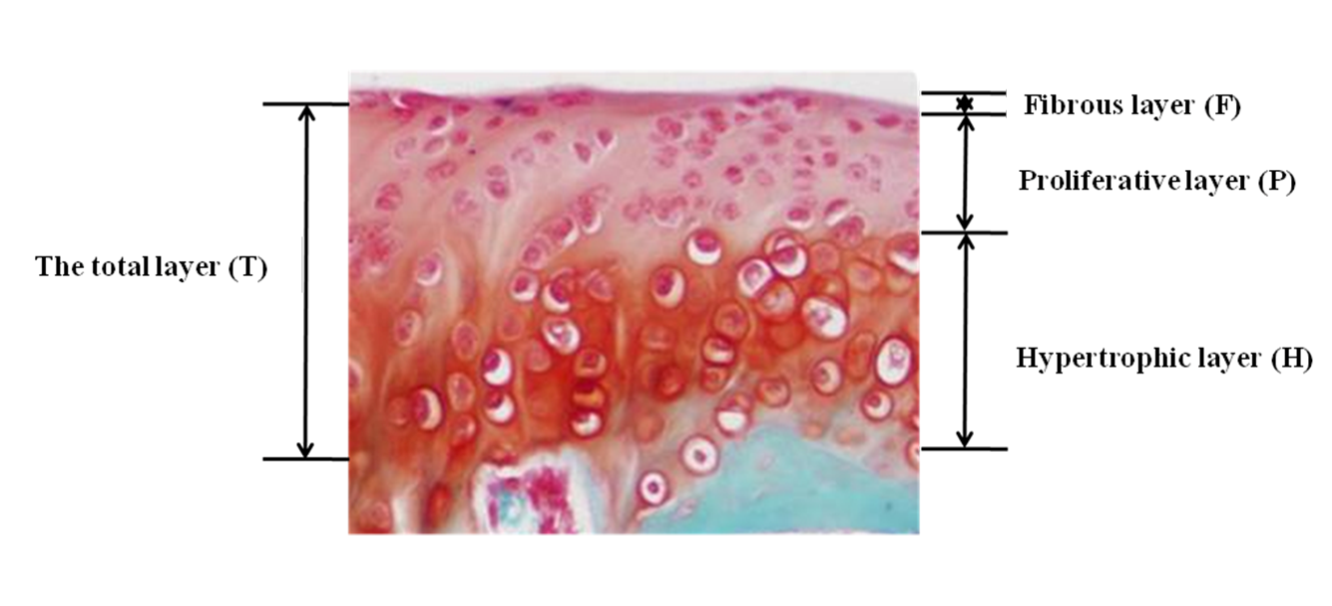


**Supplementary Figure 5**. The condylar cartilage was divided into 3 layers from outside to inside: F: the fibrous layer; P: proliferative layer; H: hypertrophic/calcified cartilage layer; T: the total layer.

**Supplementary Table 1.** The primer sequences of MMP-3, MMP-13, leptin and glyceraldehyde-3-phosphate dehydrogenase (GAPDH) used for qRT-PCR.

| PCR primers | Forward sequences  Reverse sequences |
| --- | --- |
| GAPDH | F：5'-GCAAGTTCAACGGCACAG-3'  R：5'-CCAGTAGACTCCACGACAT-3' |
| MMP-3 | F：5'-TGGGAAGCCAGTGGAAATG-3' R：5'-CCATGCAATGGGTAGGATGAG-3' |
| MMP-13 | F：5'-CTGACCTGGGATTTCCAAAA-3' R：5'-ACACGTGGTTCCCTGAGAAG-3' |
| leptin | F：5'-GGAAGCCTCGCTCTACTCCA-3'  R：5'-GAATGTCCTGCAGAGAGCCC-3' |

**Supplementary Table 2**. A semi-quantitative scoring system for long-term ostoarthritic damages in cartilage.

| **Grade** | **Osteoarthritic damage** |
| --- | --- |
| 0 | Normal |
| 0.5 | Loss of Safranin-O without structural changes |
| 1 | Small fibrillations without loss of cartilage |
| 2 | Vertical clefts down to the layer immediately below the superficial layer and some loss of surface lamina |
| 3 | Verical clefts/erosion to the calcified cartilage extending to <25% of the articular surface |
| 4 | Vertical clefts/erosion to the calcified cartilage extending to 25-50% of the articular surface |
| 5 | Vertical clefts/erosion to the calcified cartilage extending to 50-75% of the articular surface |
| 6 | Vertical clefts/erosion to the calcified cartilage extending >75% of the articular surface |
